# Complete genome sequence of *Cellulomonas* sp. GTC 20042 isolated from human bile

**DOI:** 10.1128/mra.01117-25

**Published:** 2026-01-12

**Authors:** Jun Yonetamari, Masahiro Hayashi, Yoshinori Muto, Tetsuya Iida, Kaori Tanaka

**Affiliations:** 1 United Graduate School of Drug Discovery and Medical Information Sciences, Gifu University 520344 , Gifu City, Gifu, Japan; 2 Division of Clinical Laboratory, Gifu University Hospital 476117, Gifu, Japan; 3 Division of Anaerobe Research, Life Science Research Center, Gifu University 201111, Gifu City, Gifu, Japan; 4 Institute for Glyco-core Research iGCORE, Gifu University 12785, Gifu City, Gifu, Japan; 5 Gifu University Center for Conservation of Microbial Genetic Resource, Gifu, Japan; 6 Pathogenic Microbes Repository Unit Research Institute For Microbial Diseases, Osaka University 34822, Suita, Osaka, Japan; University of Maryland Baltimore, Baltimore, Maryland, USA

**Keywords:** *Cellulomonas*, genome analysis

## Abstract

The genus *Cellulomonas* comprises aerobic, Gram-positive, rod-shaped bacteria commonly present in the soil and aquatic environments. Herein, we report the isolation of *Cellulomonas* sp. GTC20042 from human bile and the complete genome sequence of this strain, consisting of a 4,145,753-bp circular chromosome.

## ANNOUNCEMENT

*Cellulomonas*, proposed by Bergey et al. (1), includes aerobic, Gram-positive rods, mostly isolated from soil (2–4), with rare human sources (5). In 2021, *Cellulomonas* sp. GTC20042 was isolated from human bile using sheep blood agar plates (Kyokuto Pharmaceutical Industrial Co., Ltd., Tokyo, Japan), incubated at 37°C for 48 h under aerobic conditions. The strain was maintained under the same conditions.

The sample was cultured aerobically on Vitalmedia sheep blood agar plates (Kyokuto) and incubated at 37°C for 48 h. Genomic DNA was extracted from the bacterial pellet using Quick Taq HS DyeMix (TOYOBO Co., Ltd., Osaka, Japan). The cells were suspended in 100 μL of distilled water, heated at 100°C for 10 min, and centrifuged at 15,000 rpm for 5 min. The genomic DNA-containing supernatant was used for all genome analyses.

The complete genome of the isolate was sequenced using a combination of long- (Oxford Nanopore Technologies [ONT], Tokyo, Japan) and short-read sequencing (Illumina, San Diego, CA, USA) (6–8). For long-read sequencing, genomic DNA (1,000 ng) was treated with the Short Read Eliminator XS (Circulomics, Japan Genetic) to remove fragments below 10 kb. After end-repair and a second SRE XS treatment, the library was constructed using the Native Barcode Sequencing Kit (SQK-NBD114-24; ONT) without mechanical DNA shearing. The final library was purified using AMPure XP beads. Sequencing was performed using a PromethION 2i instrument with a FLO-PRO114M flow cell, and data were base-called in super accuracy mode using Dorado v7.2.14 implemented in MinKNOW v23.11.8 (all by Oxford Nanopore Technologies; https://nanoporetech.com). The run generated 2,761,482 raw reads with an N50 read length of 11,797 bp (Table 1). The raw long reads were trimmed and quality-filtered using NanoFilt v2.7.1 (7) with the parameters “-q 10 –headcrop 50 -l 1000.” For short-read sequencing, the Illumina DNA Prep (M) Tagmentation Kit (Illumina) was used for library construction, followed by 2 × 151-bp paired-end sequencing on the NovaSeq 6000 platform (Illumina). Raw short reads were processed using fastp v0.20.1 (6) with the parameters “-q 30 -n 20 -t 1 -T 1.” The short-read and mean long-read sequence qualities were assessed using fastp and NanoPlot v1.32.1, respectively (7). High-quality short (>92% bases >Q30 on average) and long (mean read quality: 14.3) reads were assembled using Unicycler v0.4.8 with default settings (8). Circular contigs generated by Unicycler were automatically trimmed for overlapping ends, and the chromosomes were rotated to start at the *dnaA* gene when detected, as part of the default pipeline (8). The assembled contig graph was visualized and assessed for integrity using BlobTools v1.0 (9). Genome completeness and contamination were evaluated using CheckM v1.2.2 (10), and functional annotation was performed using DFAST (11). Default parameters were used for all software unless otherwise specified.

**TABLE 1:** Complete genome sequence information of *Cellulomonas* sp. GTC20042 isolated from human bile

	Strain name
Parameter	*Cellulomonas* sp. GTC20042
Illumina sequencing^*a*^	
No. of reads	2,761,482
Size (bp)	408,271,901
Avg coverage (×)	91
DRA accession no.	DRR720122
ONT sequencing^*a*^	
No. of reads	104,041
Size (bp)	694,823,651
Average read length (bp)	10,144
Average coverage (×)	119
N50	11,797
DRA accession no.	DRR720121
Assembly	
Estimated genome completeness (%)^*b*^	93.66
Estimated genome contamination (%)^*b*^	3.93
Genome structure	1 chromosome
DDBJ/GenBank accession no.	AP043678
Genome size (bp)	4,145,753
GC content (%)	
(chromosome/plasmid name)	74.0
No. of coding sequences^*c*^	3,826
Number of rRNAs^*c*^	9
Number of tRNAs^*c*^	48
Number of CRISPRs^*c*^	2

^*a*^ DRA, DNA DataBank of Japan (DDBJ) Sequence Read Archive.

^*b*^ Determined using CheckM.

^*c*^ DFAST, DDBJ Fast Annotation and Submission Tool.

The taxonomic identification of *Cellulomonas* sp. GTC20042 was performed using GTDB-Tk v2.4.0 (12), classifying the strain within the genus *Cellulomonas*.

BLASTn analysis showed no identical species. The genome-derived 16S rRNA gene was most similar (98.4%) to *Cellulomonas pakistanensis* JCM 18755 (accession number: AB618146), suggesting this is a novel *Cellulomonas* species.

## Data Availability

The complete genome sequence of *Cellulomonas* sp. GTC20042, isolated from human bile, has been deposited in the DNA DataBank of Japan (DDBJ; https://www.ddbj.nig.ac.jp/index.html) under the following accession number: AP043678. Raw sequence data for GTC20042 have been deposited in the DDBJ Sequence Read Archive under the accession numbers DRR720121 and DRR720122. This study was performed in accordance with the Declaration of Helsinki.
